# Consensus criteria for sensitive detection of minimal neuroblastoma cells in bone marrow, blood and stem cell preparations by immunocytology and QRT-PCR: recommendations by the International Neuroblastoma Risk Group Task Force

**DOI:** 10.1038/sj.bjc.6605029

**Published:** 2009-04-28

**Authors:** K Beiske, S A Burchill, I Y Cheung, E Hiyama, R C Seeger, S L Cohn, A D J Pearson, K K Matthay

**Affiliations:** 1 Department of Pathology, Oslo University Hospital, N-007 Oslo, Norway; 2 Leeds Institute of Molecular Medicine, St James's University Hospital, Leeds LS9 7TF, UK; 3 Department of Pediatrics, Memorial Sloan-Kettering Cancer Center, New York, NY 10065, USA; 4 Department of Pediatric Surgery, Hiroshima University, Hiroshima 734-8551, Japan; 5 Department of Pediatrics, Keck School of Medicine, Los Angeles, CA 90027, USA; 6 Department of Pediatrics, The University of Chicago, Chicago, IL 60637, USA; 7 Section of Paediatrics, Institute of Cancer Research, Royal Marsden Hospital, Surrey SM2 5PT, UK; 8 Department of Pediatrics, University of California San Francisco School of Medicine, San Francisco, CA 94143-0106, USA

**Keywords:** neuroblastoma, INRG, minimal disease, immunocytology, QRT-PCR

## Abstract

Disseminating disease is a predictive and prognostic indicator of poor outcome in children with neuroblastoma. Its accurate and sensitive assessment can facilitate optimal treatment decisions. The International Neuroblastoma Risk Group (INRG) Task Force has defined standardised methods for the determination of minimal disease (MD) by immunocytology (IC) and quantitative reverse transcriptase-polymerase chain reaction (QRT-PCR) using disialoganglioside G_D2_ and tyrosine hydroxylase mRNA respectively. The INRG standard operating procedures (SOPs) define methods for collecting, processing and evaluating bone marrow (BM), peripheral blood (PB) and peripheral blood stem cell harvest by IC and QRT-PCR. Sampling PB and BM is recommended at diagnosis, before and after myeloablative therapy and at the end of treatment. Peripheral blood stem cell products should be analysed at the time of harvest. Performing MD detection according to INRG SOPs will enable laboratories throughout the world to compare their results and thus facilitate quality-controlled multi-centre prospective trials to assess the clinical significance of MD and minimal residual disease in heterogeneous patient groups.

The clinical course of neuroblastoma (NB) varies greatly in individual children depending on age, stage, histology and a number of genetic features (Brodeur, 2003; Maris *et al*, 2007). It is anticipated that the prognostic and predictive significance of such features will allow the identification of those children at greatest risk of relapse and poor outcome, leading to improved stratification for treatment and increased survival. The clinical significance of different prognostic factors is best assessed in large, cooperative, quality-controlled multi-centre prospective clinical trials. However before inclusion into such studies, children must be staged according to consensus risk criteria. One of the most important hallmarks of aggressive high-risk disease in children with NB is the dissemination of disease to the bone marrow (BM). The accurate detection of this disease is fundamental to the staging of children at diagnosis, this staging defining the initial treatment course. To guarantee consistency in the results collected in different laboratories, prognostic factors and metastatic disease should be evaluated by applying consensus standard operating procedures (SOPs) in all participating institutions.

The goal of the International Neuroblastoma Risk Group (INRG) Task Force is to achieve an international consensus on SOPs to improve and standardise management of children with NB. Established in 2004, the INRG Task Force constitutes experts across the different disciplines recruited from the major cooperative groups in America, Europe and Japan, as well as investigators from Australia and China. Four committees have been convened. The statistical, surgical and biological committees developed the INRG Risk Classification based on the statistical analysis of relevant clinical, histological and genetic factors (Cohn *et al*, 2009) and the INRG Staging System (INRGSS) based on clinical criteria and image defined risk factors (Monclair *et al*, 2009). Consistent with the International Neuroblastoma Staging System (INSS) and the International Neuroblastoma Response Criteria (Brodeur *et al*, 1988, 1993), the INRGSS (Monclair *et al*, 2009) proposes to evaluate BM for metastatic disease at diagnosis and following treatment.

For the purpose of initial staging at diagnosis, morphological investigations by cytological screening of bilateral BM aspirates and histological assessment of bilateral core biopsies are recommended. The INRGSS defines a BM infiltration of 10% tumour cells as the critical level to distinguish between those patients with stage M and MS metastatic disease, which can be achieved using conventional cytology. However, cytological and histological techniques have limited sensitivity and are unable to reliably detect minimal disease (MD) (Cheung *et al*, 1997). Furthermore, conventional cytomorphology and histology do not give an exact quantification of infiltrating tumour cell number. Therefore, for most children during or after completion of therapy, more sensitive methods for the detection of minimal residual disease (MRD) are needed. One of the most important tasks for the fourth committee, the Metastatic Disease Committee, has been to define common methodologies for the sensitive and accurate detection of MD and MRD in BM aspirates, peripheral blood (PB) and peripheral blood stem cell (PBSC) harvest. The two methods that have been systematically standardised are immunocytology (IC) targeting single tumour cells and ribonucleic acid (RNA)-based reverse transcriptase-polymerase chain reaction (RT–PCR).

## 

### Single-cell-based methods for detection of MD

Increased sensitivity and accurate quantification of the number of infiltrating tumour cells may be achieved by single-cell-based analytical methods, for example IC and flow cytometry (FC). Both these methods are dependant on the availability of antibodies to tumour-associated cell-surface and intracellular antigens that ideally should be expressed on all target NB cells.

### Antigens

A variety of neuroectodermal antigens have been applied for immunocytological and flow cytometric detection of NB cells (reviewed in Beiske *et al*, 2005). Many NB-associated antigens are inappropriate for detection of MD and MRD because of their heterogeneous expression in individual tumours or co-expression by normal cells (Sugimoto *et al*, 1988). In contrast, disialoganglioside G_D2_ is reported to be homogeneously and strongly expressed on neuroblastic tumours (Schulz *et al*, 1984; Cheung *et al*, 1985; Shochat *et al*, 1988), but not on normal haematopoietic cells (Cheung *et al*, 1986) and is therefore regarded as ideal for specific and sensitive detection of MD and MRD by IC and FC (Warzynski *et al*, 2002, Swerts *et al*, 2004).

### Immunocytology

The specific binding of antibodies to NB cells on cytospins or smears has been visualised by either cytochemical (Beck *et al*, 1988) or fluorescent (Favrot *et al*, 1986) detection method.

For immunocytochemical detection, alkaline-phosphatase-based techniques are preferred to those using horseradish peroxidase to avoid false-positive rates due to the abundant expression of the latter enzyme in normal BM cells. The light microscopic evaluation of immunocytochemical results allows a detailed cytomorphological study of immunopositive and -negative cells. This is important to distinguish between NB cells and haematopoietic cells that may have taken up the antigen, for example macrophages. Moreover, the chromogenic label of positive cells is stable over time, facilitating sample review in cooperative groups of experts with the goal of defining standard criteria for evaluation (Swerts *et al*, 2005), quality control and training of less experienced colleagues. Establishing immunocytochemistry is relatively inexpensive because it does not require any specific equipment beyond a conventional light microscope. Disadvantages of immunocytochemistry include restrictions regarding multi-parameter analyses. Owing to the chemical nature and size of chromogens, only two separate markers, preferably located in different cellular compartments, may be detected at the same time. Subsequent analysis with fluorescent markers is not possible when alkaline phosphate-driven substrate reactions have been used because the most commonly involved chromogens display strong autofluorescence.

Fluorescence-based IC offers advantages and disadvantages differing from those listed above. Fluorochromes are small, chemically inert molecules representing superior markers for multi-parameter analyses. Thus, two or more differentially labelled antibodies, for example directed against neuroblasts and haematopoietic cells, can be applied simultaneously to increase the specificity of the immunocytological assay (Combaret *et al*, 1989). However, cytomorphological details are difficult to recognise using a fluorescent microscope. In contrast to the standardisation that has been achieved with immunocytochemistry (Swerts *et al*, 2005), cytomorphological criteria for distinguishing immunofluorescence-labelled neuroblasts from falsely positive haematopoietic cells have never been defined and standardised. Moreover, fluorescent signals are too weak to be reviewed in a multi-headed microscope. This implies that the decision on whether neuroblasts are present in a given sample will be made by only one observer, thereby increasing the risk from subjective bias. Owing to fading, fluorescent signals need to be documented by images that do not always offer the comprehensive information with respect to label distribution and intensity. These features hamper the quality control of fluorescence-labelled samples. Fluorescence-based immunocytological detection methods are therefore not considered to be an appropriate tool for sample review, standardisation of evaluation criteria and training of observers.

### Fluorescence-based IC and fluorescence *in situ* hybridisation (FISH)

Similar to the simultaneous application of two or more antibodies, the specificity of immunocytological assays can be enhanced by subsequent performance of inter-phase cytogenetic investigations (eg FISH) targeting tumour-specific aberrations in infiltrating NB cells. In cooperation with an IT company, a European laboratory working on MD in NB developed a specific software facilitating the automated screening, imaging and counting of fluorescence-positive events in a cytological sample after staining with a fluorochrome-labelled NB-specific antibody (Méhes *et al*, 2000). The images need to be assessed by an observer who decides whether a fluorescent cell represents a neuroblast. In addition, the software records the positions of fluorochrome-positive cells on the slide and can move the microscope stage to re-localise positive cells for direct assessment in the microscope. Moreover, doubtful cells may be targeted by FISH, re-localised and recorded by the software, and evaluated by the observer either on images or in the microscope (Méhes *et al*, 2001). Though representing an attractive tool for validation of immunocytological results, the system is rarely available among laboratories analysing samples from children with NB for MD. This most likely reflects the acquisition costs that are rather high, in part reflecting the limited access to software that is only available together with a fluorescence microscope through a single company. The analysis is at least initially more labour intensive than manual evaluation of samples by IC because the observer must build a classifier comprising detailed criteria for automated picking and imaging fluorescent events. Although sample evaluation is eased by automated counting, recording and re-localisation of fluorescent cells, the decision on whether an individual cell is a tumour cell still depends on the skills of the individual observer. This is likely to be most critical in suboptimal preserved clinical samples. Moreover, it is not always possible to unequivocally document antigen expression and low-copy genetic gains and losses on two-dimensional images. These restrictions impede the review and quality control of fluorescent data in a cooperative task group as pointed out previously.

### Flow cytometry

Flow cytometry has been successfully applied by several groups as a tool for MD analysis in NB patients (reviewed in Beiske *et al*, 2005). In contrast to IC, the analysis is performed on suspended cells, which in addition to the expression of specific antigens are analysed for size and granularity. No other cytomorphological features can be recorded. All the advantages described for fluorescence-based IC also apply to FC. The simultaneous application of differently labelled antibodies against tumour cells and normal cells secures a high specificity of the analysis. Other advantages include a fast sample throughput, results being provided within a few hours. A positive result is defined as a cluster of at least 10–20 flow cytometric events in a dot plot (Campana and Coustan-Smith, 2002) and it does not depend on the cytomorphological, immunological or cytogenetic skills of the observer. However, FC cannot be regarded as a completely objective technique because the correct distinction between a few positive events and a high background depends on the observer's personal experience with the instrument and the applied antibodies.

### Sensitivity of single-cell-based assays

The requirement for at least 10–20 events to call a sample positive clearly limits the sensitivity of flow cytometric analyses because it means that the sensitivity can not exceed 1 in 10^4^ cells if only 2 × 10^5^ cells are available for investigation. Likewise, to reach a sensitivity of 1 in 10^5^ cells, 1 to 2 × 10^6^ cells are required for analysis. This is not always feasible especially when examining post-chemotherapy samples. Thus, the sensitivity of flow cytometric analyses depends both on the number of investigated cells and the presence of at least 10–20 cells expressing the correct immunophenotype. In most clinical settings this will reach 1 in 10^4^ to 10^5^ cells.

In contrast, the sensitivity of immunocytological investigations solely depends on the number of investigated cells because clustering of positive cells is not required. Several reports have demonstrated the high sensitivity (1 tumour cell in 10^4^ to 10^6^ normal cells) of immunocytological assays in BM (Cheung *et al*, 1986; Favrot *et al*, 1986; Gussetis *et al*, 1989), BM autograft (Combaret *et al*, 1989) and PB samples (Faulkner *et al*, 1998). Swerts *et al* (2004) compared IC applying only one anti-G_D2_ antibody to FC involving five differently labelled antibodies against G_D2_, CD9, CD81 and CD56 on tumour cells and against CD45 on haematopoietic cells and concluded that the sensitivity of the flow cytometric assay was about 10 times lower if equal amounts of cells were analysed.

Although some immunocytological studies indicate that the level of BM infiltration is associated with outcome in children with stage 3 and 4 NB (Moss *et al*, 1991; Seeger *et al*, 2000), thus far it is not known what level of MD is clinically relevant. Therefore which methodology will be most suitable for the detection of this disease is not clear. However, it is crucial to be aware of the differential sensitivity of methods when low numbers of tumour cells are expected in a clinical sample. Information about sensitivity is also crucial when studies are designed to identify MD levels of clinical relevance because only MD results collected at similar levels of sensitivity are comparable to each other. Therefore, the number of cells examined must be included in each report.

### Nucleic-acid-based detection of MD

The amplification of tumour-specific or tumour-associated mRNA by RT–PCR has made an enormous impact on the sensitivity and specificity of tumour cell detection, allowing the accurate, objective detection of a single tumour cell in up to 1 × 10^6^ normal cells. This technique has been used to detect MD and MRD in a number of different cancers where target mRNAs expressed in tumour cells but not in the cells of the compartment to be studied (eg BM, PB, PBSC) have been identified. Importantly like cell-based methods, the presence of tumour-specific or tumour-associated mRNA is thought to reflect the presence of the disseminating tumour cell or cells that may have the capacity to metastasise, and consequently may more closely reflect active disease status. This is in contrast to the detection of circulating free nucleic acid in plasma, serum or urine that may be useful in cancer detection, prognostication and monitoring (Taback and Hoon, 2004; Zeerleder, 2006; O'Driscoll, 2007) but reflects tumour turnover and mass.

### Optimal target selection for detection of MD by RT–PCR

Polymerase chain reaction-based amplification of tumour-specific abnormalities to detect MD has been most powerful in haematological malignancies where consistent, well-characterised translocations have been described. These studies have resulted in the introduction of new interventions to target this disease with the expectation of improved patient outcome (Faderl *et al*, 1999; Foroni *et al*, 1999; Hochhaus *et al*, 2000; Roman *et al*, 2000; Campana *et al*, 2001). Amplification of known tumour-specific gene rearrangements has also provided a powerful tool to detect potential significant MD in solid cancers including those of the Ewing's sarcoma family of tumours (de Alava *et al*, 1998; Zoubek *et al*, 1998; Schleiermacher *et al*, 2003; Avigad *et al*, 2004), alveolar rhabdomyosarcoma (Thomson *et al*, 1999; Athale *et al*, 2001; Gallego *et al*, 2006) and desmoplastic small round cell tumours (Athale *et al*, 2001).

Unfortunately for most solid tumours, tumour-specific gene abnormalities that can be exploited to detect MD have not been identified. In these cases amplification of tumour-associated wild-type mRNA by RT–PCR has been used (Burchill and Selby, 1999, 2000). Optimal detection of MD or MRD using RT–PCR for wild-type mRNAs requires the identification of a target mRNA that is expressed in the target tumour cells but not in normal haematopoietic cells or cells within the compartment to be studied. Ideally a target for the detection of MD by RT–PCR will not only be cell specific, it will also have a long half-life and expression will be unaffected by chemotherapeutics, allowing an assessment of MRD throughout treatment and the disease course. A tissue-specific wild-type mRNA should also be encoded by a gene with introns so that primers for amplification by PCR can be designed across exon–exon junctions, to selectively amplify cDNA generated from mRNA and limit any false positives generated from amplification of contaminating genomic DNA. The identification of suitable target mRNAs is one of the primary challenges for successful application of RT–PCR to detect MD in most solid cancers.

### Target selection in NB

The application of RT–PCR to detect MD in children with NB has been studied by several groups, and a number of different target mRNAs have been explored. Because catecholamines are produced by NB, the mRNA for the first enzyme in the catecholamine synthesis pathway tyrosine hydroxylase (TH) was used in initial proof-of-principle experiments. Using TH as a target detection of 1 NB cell in 10^6^ normal cells is frequently reported. Although other targets for the detection of NB cells by RT–PCR have been described, TH mRNA is currently the single most widely evaluated target (Burchill *et al*, 1994; Miyajima *et al*, 1995; Gilbert *et al*, 1997; Cheung and Cheung, 2001; Tchirkov *et al*, 2003; Ito *et al*, 2004; Ootsuka *et al*, 2008; Träger *et al*, 2008). Clinically significant disease has been detected in PB (Burchill *et al*, 1995, 2001a; Kuroda *et al*, 1997; Shono *et al*, 2000; Cheung *et al*, 2004) and BM (Miyajima *et al*, 1996; Kuroda *et al*, 1997; Shono *et al*, 2000; Fukuda *et al*, 2001; Horibe *et al*, 2001) samples from children with NB at diagnosis, on therapy, during follow-up and at relapse by RT–PCR for TH. Furthermore, this technique has been used to detect NB cells in PBSC from children with high-risk disease (Miyajima *et al*, 1996; Burchill *et al*, 2001b; Corrias *et al*, 2006). The success of TH as a target is in part attributed to the stability of the mRNA in haemopoietic compartments and its ubiquitous expression in NB cells; even those rare NB cells that do not secrete catecholamines express TH mRNA. Furthermore, most studies suggest that TH mRNA is absent or rarely expressed in normal PB or BM (Burchill *et al*, 1995; Cheung and Cheung, 2001; Träger *et al*, 2008; Viprey *et al*, 2008). Despite this literature the clinical utility of RT–PCR for TH mRNA remains unclear, reflecting the small number of patients studied, absence of quality control across studies, lack of uniform methodology for sample collection, processing and inconsistency in reporting (Riley *et al*, 2003). Therefore the clinical utility of RT–PCR for TH mRNA is currently being evaluated in a large prospective clinical trial (HR-NBL1/ESIOP; www.siopen-r-net.org), according to quality-controlled SOPs (Viprey *et al*, 2007).

### Quantitative (Q)RT-PCR

The development of quantitative reverse transcriptase-polymerase chain reaction (QRT-PCR) has increased the scope and potential for MD monitoring by RT–PCR, providing assays with a wider linear dynamic range, superior sensitivity and objective interpretation of results. These properties are enhanced by good intra- and inter-assay reproducibility, and the generation of a permanent quantitative record of the data that can be reviewed independently. Additional attractions include high-throughput capacity, speed and elimination of lengthy post-PCR handling steps, reducing the risk of potential carryover contamination.

A further obvious advantage of QRT-PCR over more traditional qualitative RT–PCR is that it allows a precise quantification of a single or multiple mRNA(s) in clinical samples. This is not necessarily a direct measure of absolute cell number because the level of a target mRNA per cell may vary, however it does allow an objective accurate measure of tumour content within and across clinical samples. This is particularly important for defining the clinically relevant level of MD and MRD at diagnosis and during disease course respectively. For accurate reporting the selection of an optimal reference mRNA against which the test mRNA(s) can be normalised is essential (Beillard *et al*, 2003; Viprey *et al*, 2007). *β*-2 microglobulin (*β2M*) is frequently selected as the standard housekeeping gene to report expression of MD in BM, PB and/or PBSC as it is stably expressed in normal haematopoietic cells (Livak and Schmittgen, 2001; Beillard *et al*, 2003). Some investigators use target-specific calibrators that allow the accurate quantitation of transcript number within a sample (Trager *et al*, 2003), although this must be reported relative to transcript number of a housekeeping gene to control for inter-assay variability (especially when different machines and/or thresholds are used). The ability to quantify the level of specific mRNAs also facilitates the development of assays with increased specificity of tumour cell detection where an ideal target has not been identified, overcoming tumour heterogeneity and low-level expression of some target mRNAs in cells of the normal compartments (Cheung and Cheung, 2001; Swerts *et al*, 2006; Cheung *et al*, 2007; Medic *et al*, 2007; Viprey *et al*, 2008). Where it has not been possible to identify specific wild-type targets for detection of disease, this can be particularly powerful based on the ability to experimentally define a cut-off to distinguish between clinically significant levels of tumour cell mRNA and levels in normal cells (Gunn *et al*, 1996; Swerts *et al*, 2006; Cheung *et al*, 2007).

## Consensus methods

Although IC and RT–PCR appear to be ideal tools for monitoring MD and MRD with a sensitivity of 1 tumour cell in 10^5^ to 10^7^ normal cells, their integration into the management of children with NB has been slow. This reflects uncertainty about clinical utility, which in part is a consequence of the small number of children studied but also inconsistency of methods and reporting making it difficult to compare results from different centres and countries. After in-depth discussions of the advantages and disadvantages of the available methods reviewed above, the members of the Metastatic Disease Committee agreed on consensus criteria for the collection and processing of haematopoietic samples and for the detection of rare NB cells in BM, PB and PBSC by IC using G_D2_ and by QRT-PCR for TH mRNA. The recommendations are reported here and include guidelines for the sampling and processing of BM aspirate, PB and PBSC and the standardised procedures for performing and reporting on IC and QRT-PCR results.

### Sampling for IC and RT–PCR and sample transport

**Figure 1:**
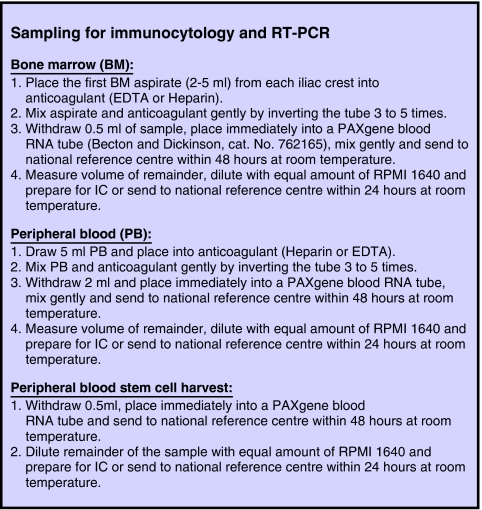
Consensus guidelines for sampling bone marrow (BM), peripheral blood (PB) and peripheral blood stem cell (PBSC) harvest for immunocytology (IC) and reverse transcriptase (RT)–PCR.

Guidelines for sampling from BM, PB and PBSC were developed and are detailed in Figure 1.

The volumes for IC and QRT-PCR are withdrawn from the same collection tube containing anticoagulant to facilitate a comparison of the sensitivity of IC and QRT-PCR for detection of MD within the same clinical sample. Sampling of two sequential BM aspirates for IC and QRT-PCR is not recommended because they are likely to differ in cellularity, reflecting dilution of multiple sequential BM aspirates with PB. Previous studies have demonstrated that RNA stabilisation and extraction efficiency using the PAXgene blood RNA tubes is unaffected by the anticoagulants EDTA or heparin. Because processing of cells for IC includes counting of mononuclear cells, the approximate number of mononuclear cells analysed by QRT-PCR can be indirectly calculated if the sample has been split in the way described and the volume analysed by QRT-PCR has been accurately measured. A direct comparison of the sensitivity of both methods, that is number of NB cells and number of TH transcripts per number of cells analysed, is not possible because the RNA for QRT-PCR is extracted from all BM cells, whereas IC analyses only the mononuclear cell population. However most importantly, the independent prognostic and predictive power of the methods can be compared using multi-variant analyses.

Some studies have shown that the number of circulating NB cells detected by IC is approximately 2 logs higher in BM than in PB (Faulkner *et al*, 1998), consistent with observations using QRT-PCR where the level of TH mRNA transcripts may be up to 3 logs greater in BM than the level observed in paired PB (Corrias *et al*, 2008). Neuroblastoma cells in PB are not currently used to assess disease status in children with NB; however this may be relevant for MD monitoring and could provide a less invasive test that is better tolerated by most children (Cheung *et al*, 2004). Consequently the INRG Metastatic Disease Monitoring Group recommends that PB in addition to BM samples should be screened for MD (Cheung *et al*, 2004).

If the BM sample size is 0.5 ml or less this sample should be taken for QRT-PCR, whereas the sample from the other iliac crest should be analysed by IC. Peripheral blood samples of 2 ml or less should be analysed by QRT-PCR given the lower frequency of circulating NB cells in blood.

#### Sample transport

Dilution of the IC sample with RPMI 1640 helps to maintain cell viability (K Beiske, unpublished observation). Samples for IC should be maintained and transported to the reference centre at room temperature. Processing samples for IC should start within 24 h of sample collection. If processing is delayed until 48 h, the sample can still be analysed, but a note should be made and included into the final report. Samples collected into PAXgene blood RNA tubes should be sent to the reference centre within 48 h at room temperature, or can be stored locally at −80°C for up to 12 months before analysis. Frozen samples can be transported to reference centres on dry ice. RNA is stable in PAXgene blood RNA tubes stored at −80°C for more than 12 months.

### IC analysis

Of the single-cell-based methods reviewed IC was chosen because it reaches a higher sensitivity than FC. Immunocytochemistry was preferred to fluorescence-based IC because screening in a light microscope facilitates the recording of cytomorphological details requisite for final evaluation. Moreover and in contrast to fluorescence-based methods, immunocytochemical staining products are permanent and can be reviewed in a multi-headed light microscope, which facilitates quality control, consensus diagnoses in difficult cases, definition of standard criteria for evaluation and training of newcomers in the field.

**Figure 2:**
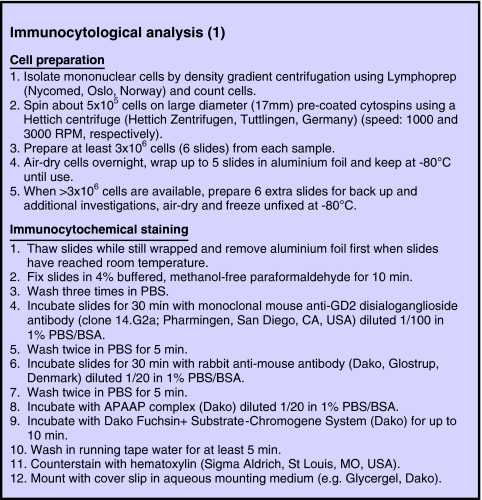
Immunocytological analysis. Consensus guidelines for cell preparation and immunocytochemical staining. Abbreviations: r.p.m., rounds per min; PBS, phosphate-buffered saline; BSA, bovine serum albumin; APAAP, alkaline phosphatase anti-alkaline phosphatase.

**Figure 3:**
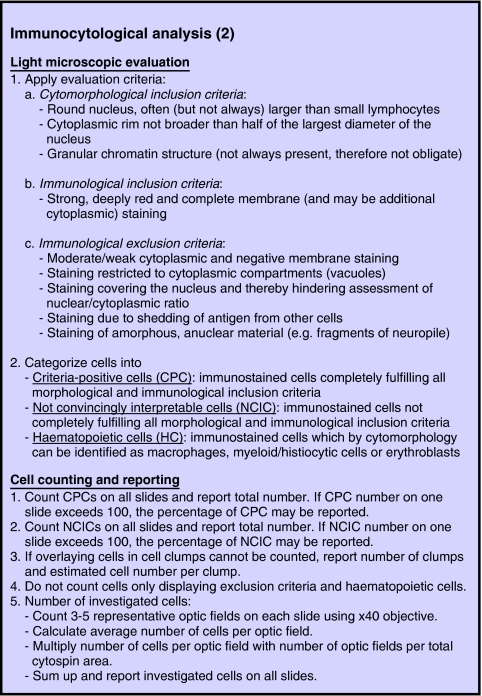
Immunocytological analysis. Consensus guidelines for light microscopic evaluation, cell counting and reporting of results.

The recommendations summarised in Figures 2 and 3 represent slight modifications of the previously published European IC consensus method (Swerts *et al*, 2005). Figure 2 describes the procedures for cell preparation and immunocytochemical staining. Step 1 of the cell preparation is skipped when handling PBSC products. If the cell number in any type of sample exceeds 3 × 10^6^, extra slides should be made and frozen without prior fixation at −80°C for back up, for example additional investigation with other markers. If frozen slides are unwrapped before thawing, the formation of condensation will make the slides stick together and possibly destroy cell morphology. Thawed slides detach without problems. For freezing, wrapping in aluminium foil is preferred to plastic boxes because boxes consume too much space and do not prevent condensation. Locally prepared cytospins can be kept frozen at −80°C, batched and sent on dry ice to the reference centre for immunostaining.

Paraformaldehyde fixation should be performed by incubating single slides on a staining bridge and not in a jar to avoid crossover contamination of single cells between individual slides. Paraformaldehyde is crucial to provide reproducible maintenance of cytomorphological details required for final evaluation (Figure 3). Acetone is an inappropriate fixative in this context because it permits swelling of cells with subsequent changes in nuclear/cytoplasmic ratio and even loss of nuclear material.

The INRG Committee for Detection of MD recommends monoclonal anti-G_D2_ antibody 14.G2a (Mujoo *et al*, 1987; BD Biosciences, Pharmingen, San Diego, CA, USA). G_D2_ was chosen as the target for IC because it is known to be strongly expressed by most neuroblastic tumours, but not by normal haematopoietic cells (Cheung *et al*, 1986) as detailed in the beginning. Presently, no other neuroblastic antigen with similar qualities is known. Schumacher-Kuckelkorn *et al* (2005) reported a heterogeneous weak or negative G_D2_ staining of infiltrating NB cells in the BM in 1 of 191 patients (0.5%) before treatment. The INRG Committee for Detection of MD recommends investigation of BM and PB already at diagnosis (see below) to identify these very rare primarily negative cases. In the same report, two other patients with originally strongly G_D2_-positive BM infiltration were found negative after anti-G_D2_ treatment, which might be explained by antigen modulation or selection of G_D2_-negative clones through therapy. Loss of G_D2_ after antibody treatment is considered to be very infrequent by others (Kramer *et al*, 1998). If anti-G_D2_ staining is found to be negative in cytomorphologically positive cases, other antibodies, for example anti-CD56, should be applied that show high sensitivity for NB cells but lower specificity than G_D2_.

For positive control of anti-G_D2_ immunostaining, cytospins from NB cell lines (eg IMR-32) should be included. Unspecific staining can be ruled out by evaluating normal BM cells in the sample, which are supposed to be completely negative for G_D2_ staining. Titrating new batches of antibodies and alkaline phosphatase anti-alkaline phosphatase (APAAP) complexes should also be performed on NB cell lines and normal BM until strong staining of NB cells but absence of staining in normal BM cells is reached.

**Figure 4:**
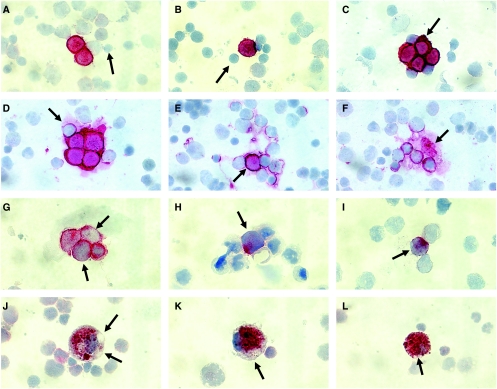
(**A**–**L**) Immunostaining of cytospins containing mononuclear cells from BM aspirates pulled from children with NB stage M. The red label shows binding of monoclonal anti-G_D2_ antibody 14.G2a visualised by a commercial alkaline phosphatase anti-alkaline phosphatase kit and Fuchsin+ substrate (Swerts *et al*, 2005). All images are taken with a × 60 dry lens and reproduced with identical magnification to enable comparison of different cell types by size. **A** and **B** show the mandatory cytomorphological and immunological criteria of neuroblasts, that is high nuclear/cytoplasmic ratio and strong G_D2_ membrane expression. A granular appearance of the chromatin is frequently, but not always recognised. Neuroblasts are usually larger than small lymphocytes (arrows). If all criteria are fulfilled, even single neuroblasts can be identified (**B**). Reliable recognition of cytomorphological and immunological criteria depends on paraformaldehyde fixation. (**C**) Typical clump formation of neuroblasts with moulding nuclei. Some tumour cells may show increased amount of cytoplasm (arrow), but the nuclear/cytoplasmic ratio should not decline below 2. Note adjacent G_D2_-negative lymphocytes. Cells that fulfil the criteria as detailed in **A**–**C** are classified as criteria-positive cells (CPC). (**D**–**F**) Shedding of G_D2_ from a clump (**D**) or a single neuroblast (arrow in **E**) on adjacent haematopoietic cells creates the impression of an almost complete (arrow in **D**) or partial membrane staining. G_D2_ may even shed from fibrillary acellular material, for example fragment of neuropile (arrow in **F**) on to normal cells. Note: no neuroblasts are identified in **F**. (**G**–**I**) Not convincingly interpretable cells (NCICs). Two of the cells in **G** (arrows) do not display a complete membrane staining. However, their nuclear size, morphology and clump formation strongly favour a neuroblastic origin. In **H**, form and size of the nucleus suggest a neuroblast, but the cell membrane is widely avoided of G_D2_. Also the cell in **I** is incompletely stained and in addition somewhat smaller than typical neuroblasts. The true nature of these cells can only be revealed by means of ancillary methods (eg FISH, single-cell PCR). Their potential clinical significance remains to be established. (**J**–**L**) Macrophages with ingested G_D2_ are often found after chemotherapy and must not be interpreted as tumour cells. Hallmarks of macrophages are a very low nuclear/cytoplasmic ratio (usually <1), a granular compartmentalisation of the antigenic material in vesicles and a negative membrane stain (arrows in **J**–**L**), although the label may even occupy the whole cytoplasm (**L**). The colour quality of ingested G_D2_ is usually not purple red as in neuroblasts (see **A**–**E**), but rather brownish due to increased iron storage after blood transfusions.

Figure 3 summarises the criteria for light microscopic evaluation, cell counting and reporting of results. Not convincingly interpretable cells (NCICs) are cells that by morphology are typical neuroblasts, but show either only weak or incomplete membrane staining, or *vice versa*, that is cells smaller than typical neuroblasts but displaying complete or incomplete membrane staining. Not convincingly interpretable cells are most frequently observed in samples taken after treatment and often colocalised with typically stained neuroblasts. They might represent tumour cells which are degenerated after chemotherapy and should be registered and reported separately to establish if they have the same prognostic impact as those cells fulfilling all inclusion criteria. Examples of convincingly interpretable cells (CPCs), false positivity of normal cells due to antigen shedding, a well-known phenomenon in NB (Ladisch *et al*, 1987), NCICs and normal macrophages having ingested G_D2_ are presented in Figure 4.

### QRT-PCR analysis

Quantitative reverse transcriptase-polymerase chain reaction for tissue-specific mRNAs provides an objective assay for the detection of MD and MRD with a numeric readout across a wide linear dynamic range. However the clinical benefit of QRT-PCR for detection of disease will most likely come from the sensitivity and specificity with which low-level disease (<10%) is detected. To minimise intra- and inter-assay variability across different laboratories, SOPs for the optimal analysis and reporting of MD detected by QRT-PCR for TH mRNA in haemopoietic compartments have been established (Viprey *et al*, 2007). These SOPs have been adopted by the INRG Metastatic Disease Committee and are summarised below.

As with all PCR-based assays cautions are taken to avoid contamination of samples; RNA is extracted from clinical samples in a designated RNA room, RT–PCR is set-up and performed in a second room, and PCR is performed using the TaqMan assay (PerkinElmer, Applied Biosystems, Foster City, CA, USA), in a third room. Single-use aliquots of dNTPs, primers, probes and buffers are prepared to avoid freeze–thaw artefacts. All consumables are RNAse free, and gloves are worn to prevent the transfer of RNases onto the tubes from the hands. All clinical samples are clearly labelled with a unique identifier, catalogued and stored in a designated −80°C freezer.

RNA is extracted from PB (2 ml), BM (0.5 ml) or PBSC (0.5 ml) stabilised in PAXgene blood RNA tubes using the PAXgene blood RNA kit according to the manufacturer's instructions, with a DNAse digestion step before elution of RNA from the column. In brief, after digestion with proteinase K the stabilised haemopoietic sample is added to the RNA extraction column, the flow-through is discarded, DNAse (1350 U; 10 *μ*l) is added to the column and incubated for 15 min at room temperature. This step is to remove any contaminating DNA; we have found digestion of contaminating DNA while RNA is bound to the column removes any DNAse-dependent non-specific digestion of RNA. The material bound to the column is washed twice and eluted in buffer BR5 (company proprietary); the 40 *μ*l elution volume is passed twice through the column to maximise yield and concentration. The concentration and purity of RNA are evaluated by measuring the optical density of the RNA solution at 280 and 260 nM; the Nanodrop ND-100 (www.labtech.co.uk) allows accurate analysis of 1 *μ*l volumes and so is suitable for quantification of small precious clinical samples without the need to dilute them. RNA samples (<1 mg ml^−1^) are stored in 10 *μ*l aliquots at −80°C.

**Figure 5:**
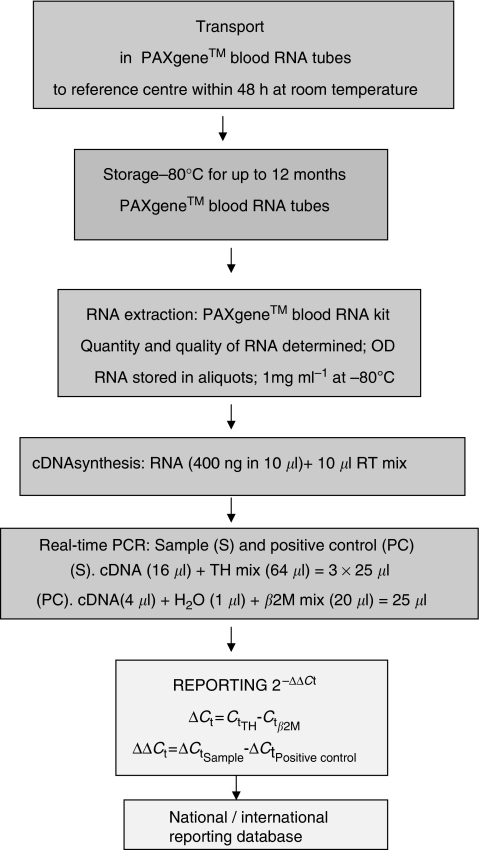
RNA is extracted and processed for detection of mRNA by QRT-PCR according to standard operating procedures.

cDNA is synthesised from 10 *μ*l of RNA (400 ng) for 1 h at 37°C, reverse transcriptase enzyme is inactivated by heating at 95°C for 5 min and amplified using the TaqMan assay as previously described (Viprey *et al*, 2007). Each sample is amplified in triplicate (3 × cDNA from 100 ng RNA) for TH mRNA and once (1 × 80 ng) for the housekeeping gene *β2M*. Negative controls of RNA with no reverse transcriptase enzyme are included to confirm the specificity of amplification from cDNA and not contaminating DNA. A positive control of cDNA generated from IMR-32 RNA (800 pg) in RNA isolated from PB from healthy volunteers (400 ng) is included to monitor any inter-assay variability. This control is also important to standardise reporting of data from clinical sample analyses that are made according to the comparative *C*_t_ method (Livak and Schmittgen, 2001) to provide an objective read-out of TH mRNA. Using this method the level of TH mRNA is normalised to expression of the housekeeping gene *β2M* and reported relative (fold difference) to the positive control sample according to the formula: 2^−ΔΔCt^, where ΔΔ*C*_t_=((*C*_t_TH−*C*_t_*β2M*)_Sample_−(*C*_t_TH−*C*_t_*β2M*)_Positive control_). Ideally the positive control should be provided by a central reference laboratory to avoid any variation attributed to differences in cell line expression of TH mRNA and sample preparation. To maintain integrity of the data, analysis and reporting from studies on clinical samples should be made independent of knowledge of other prognostic or predictive factors and patient disease status (Figure 5).

### Time points

It is recommended that analysis of BM and PB for MD should be performed at diagnosis, at the end of induction treatment (before transplantation), before treatment for MRD and at the end of therapy. Peripheral blood stem cell preparations should be analysed at the time of harvest. The demonstration of tumour cells in BM during initial staging currently remains dependant on morphology alone as detailed in the INRGSS (Monclair *et al*, 2009). It is, nevertheless, strongly recommended that BM and PB sampled at the time of diagnosis are analysed by IC and QRT-PCR to obtain baseline information on specific antigen or mRNA expression, and to investigate the potential clinical significance of MD in those children who appear not to have metastatic disease using conventional methods at diagnosis.

## Discussion

Key features of IC and QRT-PCR for detecting NB cells in BM, PB and PBSC are high specificity, sensitivity and precise quantification of cell or transcript number. For initial INRG staging, morphological assessment will be performed to identify patients with metastatic disease (INRGSS M and MS). However, to collect baseline information on the presence of specific targets in the tumour cells, IC and RT–PCR should also be performed at diagnosis. Moreover, tumour cell infiltration identified by IC or RT–PCR in the BM of INSS stage 1–3 patients (Moss *et al*, 1991) suggests that these children may benefit from upstaging and more aggressive therapeutic strategies, and could result in a re-definition of what constitutes disease free. It is crucial that further studies are performed using standardised methods for the detection of MD in a larger patient cohort. The increased sensitivity of NB detection at diagnosis might improve identification of some children for treatment with improved outcome. Furthermore, the unequivocal demonstration of MRD after chemotherapy may have important consequences for the clinical management of these children. It is conceivable that children in remission with no evidence of MRD may be spared further cytotoxic therapy, thereby reducing treatment-related complications. In children with proven metastatic disease, detection of MRD may better define response in BM and PB during successive cycles of chemotherapy. Minimal residual disease may also be used to evaluate objectively the efficacy of distinct strategies of adjuvant therapy, that is myeloablative therapy with autologous BM/PBSC rescue, or biological approaches like immunotherapy/differentiation therapy (Cheung *et al*, 2003). Finally, detection of MRD in PBSC may also be important if the re-infusion of NB cells leads to relapse (Brenner *et al*, 1994; Brenner, 1995), providing an objective method to identify harvests free of tumour cells for optimal use in transplantation and to evaluate the efficacy of purging techniques.

Both IC and QRT-PCR detect NB cells with increased sensitivity than more conventional cytology. However it is important to remember that although tumour cells in BM, PB or PBSC have the capacity to metastasise they will not necessarily go on to form secondary disease, as this is dependant on other biological processes (Eccles and Welch, 2007). It is therefore critical to evaluate the clinical significance of detecting MD or MRD in quality-controlled prospective clinical trials facilitating the interpretation of MD data in relation to patient age, stage, genetic markers and treatment modalities. The introduction of the above SOPs will allow a comparison of data between participating centres and across different studies to more efficiently evaluate the clinical significance of MD and MRD detected by IC and QRT-PCR, while also allowing a direct comparison of efficiency of treatments for MD. In such studies the limitations and advantages of IC and QRT-PCR must be considered.

Immunocytology sample preparation and immunostaining are easily standardised and reproducibly performed in routine cytological laboratories, especially if an automatic staining device is available that in addition facilitates high sample throughput. The correct application of cytomorphological and immunological evaluation criteria necessitates careful cytological examination by a trained investigator to ensure reproducible and comparable results. Immunocytochemically stained slides are well suited to create training sets. They may be batched after production in local laboratories and collected for central review by expert panels. The application of consensus criteria for immunological and morphological evaluation of anti-G_D2_-stained BM samples and the establishment of criteria for selecting critical samples for central review have led to a significant improvement of inter-observer concordance among the participants of a European multi-centre study (Swerts *et al*, 2005).

An important advantage of IC is the ability to quantify the number of tumour cells and normal cells in each specimen, because the total number of analysed cells defines the sensitivity of each individual investigation. To obtain a sensitivity of one tumour cell detected in 1 × 10^6^ normal cells, the analysis of 3 × 10^6^ PB and BM cells is required (Cheung *et al*, 1986). If the absolute number of tumour cells is critically important for the clinical development of metastases, it is anticipated that IC may lead to an objectively defined cell number that predicts outcome for future risk grouping and stratification for therapy.

In contrast QRT-PCR does not measure absolute tumour cell number, although the quantitative standardised detection of target mRNAs permits an accurate assessment of tumour load across samples and patients. The main advantages of QRT-PCR for the detection of MD are the simplicity with which samples are optimally collected and processed, the transferability of the assays and the objective numeric end point.

For both assays the evaluation of samples with several antibodies or for multiple mRNA species may increase the specificity of MD and MRD detection in heterogeneous samples. Marker discovery based on differential gene expression profiling, stringent sensitivity and specificity assays, and well-annotated patient samples can rapidly prioritise and identify novel MRD markers of NB (Cheung *et al*, 2008). Interestingly a combination of techniques or targets might provide not only prognostication about the clinical significance of MD but could also provide additional biologically relevant information. For example the expression of specific target mRNAs may identify profiles that are prognostic or predictive of the behaviour of the metastatic tumour cell (Wagner *et al*, 2006; Grau *et al*, 2009). Alternatively analysis using multiple complementary methods may be valuable to define prognostic/predictive groups, for example the coupling of anti-G_D2_ antibodies with antibodies against proliferation markers or with DNA probes capable of detecting genetic alterations of prognostic significance (Méhes *et al*, 2001). In contrast to methods based on extracting DNA, RNA or proteins from clinical samples, the cell-based nature of IC or of cytogenetic analyses permits monitoring possible heterogeneous expression patterns of biologically relevant markers within a population of circulating tumour cells. *In situ* hybridisation or single-cell PCR should at least be considered for samples only presenting NCICs as proposed by the SIOP European Neuroblastoma group (Swerts *et al*, 2005). However, the INRG Committee for Detection of MD regards these techniques too expensive and specialised to be recommended as standard facilities in all laboratories working on MD in NB. Nevertheless, saving supplementary slides and RNA samples is strongly recommended because new techniques and markers will emerge in the future. The methods and targets for MD analysis proposed in this paper should not be regarded as final, but will be revised when appropriate.

Despite the increased sensitivity of IC and QRT-PCR to detect NB cells, it is clear that cells are not detected in BM or PB of all children with high-risk disease, reflecting the biological process of metastases, the limitation of analysing a small sample volume and/or possibly the heterogeneity of NB cells. To determine whether tumour heterogeneity is significant in MRD surveillance, multiple independent techniques, for example the inclusion of both IC and QRT-PCR for the assessment of MRD in each clinical sample, or the analysis of samples using multiple antibodies or amplifying for several target mRNAs are desirable (Cheung *et al*, 1998). To evaluate the impact of IC and QRT-PCR, alone and in combination, on the detection of clinically significant disease it was agreed that BM, PB and PBSC samples will be divided using a standard procedure after sampling. In the future, the utility of multiple markers for IC and QRT-PCR will be evaluated and their utility compared to the standards described in this paper. Before any single or panel of markers can be introduced as a reliable parameter for the evaluation of clinically relevant MD or MRD, their clinical significance must be demonstrated in large prospective cooperative multi-centre studies, performed according to SOPs. The methodological recommendations for the performance of IC and QRT-PCR proposed by the INRG subcommittee in this report may serve as a first step in an international effort that aims to identify and validate MRD markers for clinical application.
